# Pure mucinous carcinoma of the breast: clinicopathologic characteristics and long-term outcome among Taiwanese women

**DOI:** 10.1186/1477-7819-11-139

**Published:** 2013-06-14

**Authors:** Hsin-Shun Tseng, Che Lin, Szu-Erh Chan, Su-Yu Chien, Shou-Jen Kuo, Shou-Tung Chen, Tsai-Wang Chang, Dar-Ren Chen

**Affiliations:** 1 Comprehensive Breast Cancer Center, Changhua Christian Hospital, 135 Nanhsiao Street, Changhua 50006, Taiwan; 2 Department of Environmental Engineering, National Chung-Hsing University, Taichung 40227, Taiwan; 3 School of Medicine, Chung Shan Medical University, Taichung 40201, Taiwan; 4 Department of Surgical Medicine, Erlin Branch of Changhua Christian Hospital, Changhua 52665, Taiwan; 5 Department of Pharmacology, Changhua Christian Hospital, Changhua 50006, Taiwan; 6 Department of Surgery, College of Medicine, National Cheng Kung University, Tainan 70101, Taiwan

**Keywords:** Mucinous carcinoma, Infiltrating ductal carcinoma, Prognosis, Hormone receptor

## Abstract

**Background:**

Pure mucinous carcinoma (MC) is found in about 3.5% of all newly diagnosed breast cancer patients in Taiwan. MC is a relatively rare malignancy of breast cancer, and its nature, behaviors, treatment pattern and long-term follow-up are not well understood. The study aimed to investigate the incidence rate, treatment patterns, and prognostic factors of MC of the breast and the clinical long-term outcomes compared with infiltrating ductal carcinoma not otherwise specified (IDC) in the middle and south Taiwanese women.

**Methods:**

Data from 93 patients with breast MC were retrospectively reviewed and the clinicopathologic characteristics and survival status were compared with those of 2,674 patients with IDC.

**Results:**

The expression of hormonal receptor was higher in MC than those in IDC (*P* <0.001). MC also demonstrated lower Her2/neu gene over-expression (*P* = 0.023), less axillary lymph node involvement (*P* <0.0001), lymphovascular invasion (*P* <0.0001) and higher 10-year overall survival rate (*P* = 0.042), when compared with those of IDC.

**Conclusion:**

Our data confirm the less aggressive behavior of MC compared to IDC. MC showed favorable clinicopathologic characteristics in tumor grade, hormone receptor status and lymph node involvement in the middle and south Taiwanese women.

## Background

Breast cancer is the most common cancer in female patients and is the leading cancer type in Taiwan. The majority of breast cancers are invasive ductal and lobular carcinomas. Pure mucinous carcinoma (MC) is a relatively rare histological type of breast cancer and accounts for 1% to 7% of all invasive mammary cancers [1,2]. MC of the breast is characterized by a mucinous morphology in over 90% of the tumor. This subtype of patients has some features that differ from those of patients with infiltrating ductal carcinoma not otherwise specified (IDC). MC has a lower incidence of nodal involvement, favorable histological grade and higher estrogen receptor (ER) and progesterone receptor (PR) [3] expression, and usually occurs in women aged over 60 years [4]. The diagnosis of MC of the breast has a favorable prognosis and has shown excellent long-term survival in a previous study [5].

Because of its rarity, most studies on MC of the breast have fewer case numbers, and information on MC after long-term follow up is limited. There is still controversy about the prognostic factors of MC of the breast. The literature recommends that this subtype of cancer should be treated less aggressively than IDC. However, the evidence of previous studies is weak due to the small sample size and short-term follow up.

The health policy for breast cancer screening has evolved since 1995 in Taiwan. With the increasing incidence of breast cancer, the incidence of patients with a sparse tumor has also increased. Therefore, greater understanding about these rare tumors is urgent. The aims of this study were to investigate the incidence rate, treatment patterns, and prognostic factors of MC, and the long-term clinical outcomes compared with IDC in middle and south Taiwanese women.

## Methods

In this study, we retrospectively reviewed the data on 2,767 breast cancer patients treated at Changhua Christian Hospital and National Cheng Kung University Hospital, Taiwan, between November 1996 and August 2009. Of this group, 93 patients who presented with MC and 2,674 with IDC were identified from the cancer registry database. The data before 1996 were excluded due to incomplete information on hormone receptor status. This study was approved by the institutional review board and ethics committee of our hospital. The medical records of patients with MC were reviewed. MC of the breast was defined in our hospital as having a mucin component of more than 90% of the lesion. The American Joint Committee on Cancer (AJCC) staging system was used in this study. The clinical baseline data, including demographic characteristics (for example, age), tumor features (for example, stage, tumor size, nodal involvement, histological grade, lymphovascular invasion, ER, PR and HER2 status) and treatment patterns (for example, primary treatment and systemic therapy) were collected. Survival time was defined as the time from pathologic diagnosis to the most recent patient status or database update. Distant and loco-regional recurrences were defined as clinical evidence of distant and local tumor recurrence. All patients were treated according to our breast cancer guidelines (based on the National Comprehensive Cancer Network (NCCN) [3] and/or St Gallen guidelines). Overall survival and disease-free survival was calculated at 10 years of follow up. The prognostic risk of age at diagnosis, tumor size, stage, histology grade, ER, PR, and HER2 status, primary treatment and systemic therapy was calculated by multivariate and univariate analysis. The clinicopathologic characteristics and survival data of MC were reviewed and compared with those of IDC.

Analysis of variance (ANOVA) was used for the comparison of continuous variables, and categorical variables were normally tested by the χ^2^ test when appropriate. Data are expressed as the mean ± SD for continuous variables. All *P-*values were two-tailed; a *P-*value less than 0.05 was considered to indicate statistical significance. Cox logistic regression was conducted for multivariate analysis. The Kaplan-Meier method was used for cumulative survival rates, and differences in cumulative survival were assessed using the log-rank method. The SAS 9.1.3 for Windows software package was used for analysis (SAS Institute Inc., Cary, NC, USA).

## Results

From 1996 to 2009, a total of 93 patients with MC of the breast were identified in our database. At the same time, 2,674 patients with IDC were also identified. The incidence of MC of the breast in our study was 3.4% (93 of 2,767) of breast cancer cases. The clinicopathologic characteristics of all patients are summarized in Table 1. Mean age at diagnosis and tumor size was not statistically significantly different in the MC and IDC cases. The majority of MC was early stage (89%); only nine patients were at the advanced stage. Expression of the hormonal receptors, ER and PR, was higher in MC than in IDC. MC also presented with lower HER2/neu gene overexpression and less axillary lymph node involvement and lymphovascular invasion.

**Table 1:** Characteristics of patients with mucinous carcinoma and infiltrating ductal carcinoma

		**MC (n = 93)**	**IDC (n = 2,674)**	***P***
Age, years,				
	mean (SD)	49.77 (13.76)	50.68 (11.39)	0.529
	median	45.88	49.22	
	range	28, 85	15, 89	
Tumor size, cm,				0.185
	mean (SD)	3.06 (2.55)	2.69 (1.65)	
	median	2.50	2.30	
	range	0.20, 17.0	0.10, 15.0	
Tumor stage, n (%),				0.480
	T1	29 (34.12)	1123 (42.00)	
	T2	47 (55.29)	1297 (48.50)	
	T3	7 (8.24)	178 (6.66)	
	T4	2 (2.35)	76 (2.84)	
	unknown or other	8	0	
Grade, n (%),				<0.0001
	I	22 (66.67)	251 (14.13)	
	II	11 (33.33)	994 (55.97)	
	III	0 (0)	531 (29.90)	
	unknown or other	60	898	
Lymphovascular invasion, n (%),				<0.0001
	present	10 (15.38)	996 (47.41)	
	absent	55 (84.62)	1105 (52.59)	
	unknown or other	28	573	
ER status, n (%),				<0.0001
	positive	84 (90.32)	1715 (65.28)	
	negative	9 (9.68)	912 (34.72)	
	unknown or other	0	47	
PR status, n (%),				<0.0001
	positive	74 (79.57)	1555 (59.19)	
	negative	19 (20.43)	1072 (40.81)	
	unknown or other	0	47	
HER2/neu gene overexpression status, n (%),				0.023
	positive	8 (9.88)	467 (19.95)	
	negative	73 (90.12)	1874 (80.05)	
	unknown or other	12	333	
Lymph node status, n (%),				<0.0001
	positive	17 (19.77)	1144 (43.73)	
	negative	69 (80.23)	1472 (56.27)	
	not evaluated	7	58	
Primary treatment, n (%),				0.660
	mastectomy	59 (64.84)	1735 (67.27)	
	lumpectomy with RT	30 (32.97)	751 (29.12)	
	lumpectomy without RT	2 (2.20)	93 (3.61)	
	no surgery	2	95	
Systemic therapy, n (%),				<0.0001
	endocrine therapy alone	37 (44.05)	523 (21.02)	
	chemotherapy alone	6 (7.14)	603 (24.24)	
	endocrine therapy with chemotherapy	41 (48.81)	1362 (54.74)	
	none	8	186	
	unknown	1	0	
Follow up time, months				
	median	56	55	
	range	1.97-142	0.30-142	
10-year overall survival rate		0.9447	0.8611	0.042

*MC* pure mucinous carcinoma, *IDC* infiltrating ductal carcinoma, *ER* estrogen receptor, *PR* progesterone receptor, *RT* radiotherapy.

The primary treatment pattern of the MC patients was similar to that of the IDC patients. The majority of patients (65% in the MC group and 68% in the IDC group) received mastectomy. Two patients in the MC group and 95 in the IDC group did not undergo surgery. In terms of systemic therapy, more MC patients received endocrine therapy alone because more of these patients were hormonal receptor-positive and had a favorable histology grade. On the other hand, IDC patients received more chemotherapy than MC patients (79% vs 56%).

**Figure 1:**
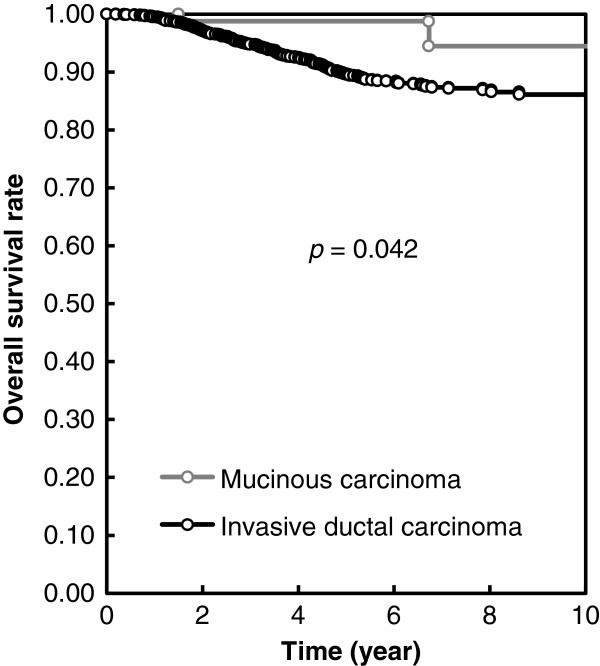
Overall survival curve of patients.

The mean follow up time for the MC and IDC patients was 56 months (range 2.0 to 142.0) and 55 months (range 0.3 to 142.0), respectively. The 10-year overall survival rate was 94.5% vs 86% for the MC and IDC patients, respectively (*P* = 0.042) (Figure 1), indicating MC had a better long-term outcome than IDC, with statistical significance; there were six disease relapses and five deaths during the period of follow up. Univariate analysis for disease-free survival and overall survival based on clinicopathologic features in the MC group revealed no significant factors for survival (Table 2). More than 80% of MC patients were hormonal receptor-positive (Table 1). In early MC breast cancer (stages I and II), the 10-year disease-free survival rate and the 10-year overall survival rate of the patients treated with hormone therapy only were both higher than those of patients treated with hormone therapy and chemotherapy, although neither was statistically significantly different (*P* = 0.529 and *P* = 0.156) (Table 3). As shown in Table 4, among the 45 patients with stage II MC, 30 patients were treated with hormone therapy plus chemotherapy and 15 with hormone therapy alone. After long-term follow up, only two deaths were noted in the hormone therapy plus chemotherapy group.

**Table 2:** Univariate analysis of disease-free survival and overall survival of patients with pure mucinous carcinoma

	**Disease-free survival**		**Overall survival**	
	**Hazard ratio**	***P***	**Hazard ratio**	***P***
Age, years	>1	0.603	<1	0.329
Tumor size, cm	<1	0.717	>1	0.999
Grade, I vs II	NA	0.998	NA	NA
Lymphovascular invasion (present vs absent)	NA	0.998	NA	0.999
ER (positive vs negative)	<1	0.139	<1	0.997
PR (positive vs negative)	<1	0.580	<1	0.343
HER2 (positive vs negative)	>1	0.122	<1	0.998
Lymph node status (positive vs negative)	NA	0.997	NA	0.998
Primary treatment (mastectomy vs lumpectomy with RT)	<1	0.996	<1	0.997
Primary treatment (mastectomy vs lumpectomy without RT)	<1	0.997	<1	0.998
Systemic therapy (endocrine therapy alone vs with chemotherapy)	>1	0.746	NA	NA

*ER* estrogen receptor, *PR* progesterone receptor, *RT* radiotherapy, *NA* not applicable.

**Table 3:** Characteristics of patients with early-stage (I and II) pure mucinous carcinoma of the breast

		**Endocrine therapy**		***P***
		**With chemotherapy (n = 41)**	**Without chemotherapy (n = 37)**	
Age, years,				<0.0001
	mean (SD)	43.85 (7.53)	58.15 (15.62)	
	median	44.54	59.54	
	range	28, 68	28, 85	
Tumor size, cm,				0.059
	mean (SD)	3.42 (2.62)	2.39 (1.93)	
	median	3.0	2.0	
	range	0.20, 17.0	0.20, 12.0	
Tumor stage, n, (%),				<0.0001
	T1	5 (13.51)	19 (52.78)	
	T2	28 (75.68)	15 (41.67)	
	Unknown or other	8	3	
grade, n (%),				1.000
	I	11 (73.33)	8 (66.67)	
	II	4 (26.67)	4 (33.33)	
	Unknown or other	26	25	
Stage, n (%)				<0.0001
	I	4 (11.76)	19 (55.88)	
	II	30 (88.24)	15 (44.12)	
Lymphovascular invasion, n (%),				0.018
	present	7 (28)	1 (3.33)	
	absent	18 (72)	29 (96.67)	
	unknown or other	16	7	
ER status, n (%),				0.112
	positive	35 (85.37)	36 (97.30)	
	negative	6 (14.63)	1 (2.70)	
PR status, n (%),				0.233
	positive	32 (78.05)	33 (89.19)	
	negative	9 (21.95)	4 (10.81)	
HER2 status, n (%),				0.199
	positive	5 (14.29)	1 (3.03)	
	negative	30 (85.71)	32 (96.97)	
	unknown or other	6	4	
Lymph node status, n (%),				0.013
	positive	11 (28.21)	2 (5.56)	
	negative	28 (71.79)	34 (94.44)	
	not evaluated	2	1	
Primary treatment, n (%),				0.056
	mastectomy	31 (77.50)	20 (54.05)	
	lumpectomy with RT	8 (20)	16 (43.24)	
	lumpectomy without RT	1 (2.50)	1 (2.70)	
	no surgery	1	0	
Follow up time, months				
	median	63	46	
	range	3, 139	9, 142	
10-year disease-free survival		87.90%	92.86%	0.529
10-year overall survival rate		88.82%	100%	0.156

*ER* estrogen receptor, *PR* progesterone receptor, *RT* radiotherapy.

**Table 4:** Characteristics of patients with stage II pure mucinous carcinoma of the breast

		**Endocrine therapy**		***P***
		**With chemotherapy (n = 30)**	**Without chemotherapy (n = 15)**	
Age, years				0.001
	mean (SD)	43.43 (6.17)	61.16 (16.89)	
	median	44.68	64.20	
	range	31.0, 57.0	28.0, 84.0	
Tumor size, cm				0.772
	mean (SD)	3.60 (2.89)	3.34 (2.43)	
	median	3.0	2.8	
	range	0.20, 17.0	2.0, 12.0	
Tumor stage, n (%),				0.545
	T1	2 (7.14)	0 (0)	
	T2	26 (92.86)	14 (100)	
	unknown or other	2	1	
Grade, n (%),				1.000
	I	9 (81.82)	3 (75)	
	II	2 (18.18)	1 (25)	
	unknown or other	19	11	
Lymphovascular invasion, n (%),				0.624
	present	4 (21.05)	1 (8.33)	
	absent	15 (78.95)	11 (91.67)	
	unknown or other	11	3	
ER status, n (%),				1.000
	positive	28 (93.33)	14 (93.33)	
	negative	2 (6.67)	1 (6.67)	
PR status, n (%),				0.695
	positive	23 (76.67)	13 (86.67)	
	negative	7 (23.33)	2 (13.33)	
HER2 status, n (%),				1.000
	positive	1 (3.70)	1 (7.14)	
	negative	26 (96.30)	13 (92.86)	
	unknown or other	3	1	
Lymph node status (%)				0.396
	positive	6 (20)	1 (6.67)	
	negative	24 (80)	14 (93.33)	
Primary treatment, n (%),				1.000
	mastectomy	23 (76.67)	11 (73.33)	
	lumpectomy with RT	7 (23.33)	4 (26.67)	
Follow up time, months				
	median	57	43	
	range	3, 139	12, 86	
10-year disease-free survival		87.69%	75%	0.637
10-year overall survival rate		88.44%	100%	0.424

*ER* estrogen receptor, *PR* progesterone receptor, *RT* radiotherapy.

## Discussion

MC of the breast is uncommon, comprising approximately 4% (range 1% to 7%) of all invasive breast cancers. MC has better prognosis (90% survival at 10 years) and higher incidence in perimenopausal and postmenopausal age groups. The incidence of MC in women under 35 years of age is only 1% [1,6-8]. MC is a slow-growing tumor, with an estimated growth rate of one third of that of IDC. MC shows fewer axillary lymph node metastases and more frequent ER expression, and has a low frequency of androgen receptor and low incidence of androgen receptor with ER and/or PR co-expression when compared to IDC [9]. Under ultrasonography, MC is commonly lobulated with homogeneous, iso-echoic and normal posterior acoustic appearances, but rarely has benign features on imaging [7,8].

The majority of MC (89%) presented at the early stages (I and II) with favorable clinicopathologic features. However, the mean age at diagnosis of MC was 49.8 years in this study, which was much younger than in a study of Western women [10]. Many previous studies have reported that MC was more frequently diagnosed in women older than 70 years. However, the incidence of breast cancer in Taiwan has increased recently. The mean age of breast cancer diagnosis (49 years) was advanced by 10 years. There was a similar trend among the patients presenting with MC. The reason for the younger age at diagnosis among Taiwanese women is not clear, although there are some explanations. First, there are some physiological differences between women in the West and the East. In Asia, the breasts of women are smaller and denser. Most MC patients presented with palpable masses and the wide use of ultrasound made it easier to diagnose earlier in our country. Second, the health policy for breast cancer screening has evolved since 1995. Taiwanese women over 45 years old undergo mammography every 2 years. As a result, more middle-aged patients were shifted from the population. Third, dietary habits and environmental and genetic factors differ from Western countries. Because of the small number of reported cases, further studies related to the environmental, genetic and pathological features of Asian women are needed.

Previous studies have reported that MC had more favorable clinicopathologic characteristics than IDC [2,4,10,11]. In our study, MC had a lower histologic grade, less lymphovascular invasion, higher hormonal receptor expression (ER and PR) and less HER2/neu gene overexpression. MC also demonstrated less axillary lymph node involvement than IDC, with a statistically significant difference. In addition, there were no significant differences in age, tumor size and pathologic stage. The 10-year survival rate of MC patients was 94.5%, which was statistically significantly better than the IDC survival rate. Our report ascertained favorable long-term outcomes for patients with MC. Previous studies also showed that MC was more frequently associated with hormone receptor expression [11,12]; this was in concordance with our study, in which the positive rates for ER and PR were 90% and 80%, respectively. Hormone receptor-positive tumors were frequently associated with a well-differentiated histology pattern and were sensitive to adjuvant hormonal therapy. The long-term prognosis for MC was better than for IDC tumors. However, some investigators showed that breast IDC with a high level of hormone receptor expression was less sensitive to chemotherapy [13-15]. MC has been proven to be a hormone receptor-rich tumor and differs in tumor characteristics, including tumor behavior and response to treatment, from IDC. A genomic study confirmed that MC is an entity distinct from IDC, and that tumor behavior characterized by genetic aberrations is a luminal phenotype [16]. The St Gallen guideline for ER-positive breast cancer recommends that chemotherapy should not be administrated if the tumor is highly endocrine-responsive and has a low risk of recurrence. When planning systemic therapy for patients with MC, we should be concerned that ER-rich tumors are more likely to benefit from tamoxifen than from chemotherapy. When adding chemotherapy to endocrine-based adjuvant therapy, the benefit of systemic chemotherapy should be considered carefully in the treatment of these slow-growing tumors.

Axillary lymph node involvement has been known as an important prognostic factor in IDC. Bae *et al*. identify that MC is shown to have better disease-free survival than IDC, but it is similar in overall survival. They find that nodal status and adjuvant therapy seem to be more significant predictors of prognosis than histologic subtype [17]. Di Saverio *et al*. use tumor size as an independent prognostic indicator, but it was less significant compared to nodal status and age in MC cases [10]. Although we did not compare the DNA stemline of MC and IDC in our study, Toikkanen *et al*. have already found that nearly all MC has a normal diploid stemline that is different from that of common ductal carcinoma. Aneuploid tumors tend to be of higher grade and stage than diploid tumors [18]. Jambal *et al*. developed a human breast cancer cell line called BCK4, that can be the unique model to study the phenotypic plasticity, hormonal regulation, optimal therapeutic interventions and metastatic patterns of MC more clearly [19].

According to the treatment guidelines, adjuvant chemotherapy is indicated for IDC patients with lymph node metastases. However, lymph node involvement was not a good prognostic factor in our study. In our univariate and multivariate analysis of disease-free survival and overall survival of patients with MC, we found no pathologic features that were significant prognostic risk factors (Table 2). Adjuvant hormone therapy was adequate if the patients were hormone receptor-positive.

Tumor size in the AJCC staging system may not be a significant factor because mucin comprises the majority of the tumor volume [11]. According to the NCCN Clinical Practice Guidelines in Oncology [3], adjuvant endocrine therapy is indicated for tumors more than 3 cm in diameter and for MC patients with axillary lymph node metastasis, adjuvant endocrine therapy plus or minus chemotherapy should be considered. Further large-scale prospective studies are urgently needed to define the therapeutic guidelines for MC breast cancer.

## Conclusions

MC is a relatively rare type of breast cancer, occurring in about 3.5% of all newly diagnosed breast cancer patients in the middle and south of Taiwan. It has been associated with a better long-term prognosis than IDC. Our data confirm the less aggressive behavior of MC compared to IDC. MC had favorable clinicopathologic characteristics in terms of tumor grade, hormone receptor status and lymph node involvement.

## Abbreviations

AJCC: American Joint Committee on Cancer; ANOVA: Analysis of variance; ER: Estrogen receptor; IDC: Infiltrating ductal carcinoma; MC: Mucinous carcinoma; NCCN: National Comprehensive Cancer Network; PR: Progesterone receptor.

## Competing interests

The authors declare that they have no competing interests.
